# A comprehensive analysis of immune infiltration in the tumor microenvironment of osteosarcoma

**DOI:** 10.1002/cam4.4117

**Published:** 2021-07-13

**Authors:** Hao Yang, Liang Zhao, Yang Zhang, Fang‐Fang Li

**Affiliations:** ^1^ Department of Orthopedics The First Affiliated Hospital of Zhengzhou University Zhengzhou China; ^2^ Department of Respiratory Medicine The First Affiliated Hospital of Zhengzhou University Zhengzhou China

**Keywords:** cancer immunotherapy, cold/hot tumors, immune infiltration, osteosarcoma

## Abstract

**Background:**

Even though immunotherapy has been an effective treatment for solid tumors, its efficacy in osteosarcoma remains sub‐optimal. It is therefore imperative to understand the complex tumor microenvironment (TME) of osteosarcoma to facilitate the development of immunotherapies against this cancer.

**Methods:**

The mRNA expression profiles of osteosarcoma tissues were downloaded from The Cancer Genome Atlas (TCGA) database. Next, the ssGSEA, MCP‐counter, CIBERSORT, and Xcell algorithm analyses were performed to characterize the tumor microenvironment of osteosarcoma tissues. The tumor tissues were divided into inflammatory and non‐inflammatory. A comprehensive assessment of immune cell infiltration in osteosarcoma tissues was then performed. Sub‐group analysis of immune cell infiltration between men and women patients with osteosarcoma was also carried out.

**Results:**

The results revealed that the infiltration of immune cells including activated B cell, activated CD8 T cell, CD56dim natural killer cell, and cytotoxic lymphocytes cells, in osteosarcoma tissues was higher in male than in female patients. Based on the infiltration profile of different immune cells, the osteosarcoma tissues were grouped into four clusters. The four clusters were further divided into hot and cold tumors. The differently expressed genes (DEGs) between cold and hot tumors were mainly associated with the activation and regulation of immune response. Additionally, a neuronal pentraxin (NPTX2) expression which was upregulated in cold tumors was found to be negatively correlated with the expression of CD8a Molecule (CD8A), Granzyme B (GZMB), and Interferon Gamma (IFNG). NPTX2 decreased CCL4 secretion. Knockdown of NPTX2 in osteosarcoma cells inhibited tumor growth and increased tumor cell apoptosis. Moreover, a prognosis prediction model of osteosarcoma was constructed and validated in patients receiving immunotherapy using external data.

**Conclusions:**

To the best of our knowledge, this is the first study to characterize the infiltration of immune cells in osteosarcoma tissues from patients receiving immune infiltration therapy.

## Graphical abstract

We comprehensively analyzed the possible mechanism of the difference in the immune infiltration of the tumor microenvironment of osteosarcoma, screened out the hub gene set that affects immune infiltration, and verified the survival.

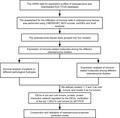

## 1 INTRODUCTION

Primary malignant tumors of the bone account for less than 0.2% of all cancers.^1^ Meanwhile, osteosarcoma is the most common primary bone cancer accounting for 19% of all bone malignancies. It is also the third most common malignancy in children and adolescents.^2^, ^3^ The search for efficient treatments for osteosarcoma has been ongoing for several decades now. For instance, from 1982 to 1984, in the Multi‐Institutional Osteosarcoma Study (MIOS), 113 patients were randomly divided into two groups. One group underwent surgery alone, whereas the other underwent surgery plus adjuvant chemotherapy. It was found that the 6‐year survival rate of patients who underwent surgery only was 11%, against 61% for patients who received both surgery plus adjuvant chemotherapy.^4^ Elsewhere, the survival rate of patients with metastatic cancers (most common in lung parenchyma and distant bones) was only about 19%–30%.^5^, ^6^ Despite the advances in cancer treatment over the past few decades, the survival rate of patients with osteosarcoma remains unsatisfactory. Immunotherapy is a recent cancer treatment option that has achieved excellent results against numerous solid tumors. However, immunotherapy against osteosarcoma has not been developed. Therefore, we explored the infiltration pattern of immune cells in osteosarcoma tissues using data in the TCGA database. The findings of this study may guide the development of osteosarcoma immunotherapy.

Infiltration of lymphocytes into tumor tissues is a phenomenon that was discovered more than 100 years ago. However, research on the relationship between immunity and prognosis began later in the 1960s.^7^ Studies have shown that the tumor microenvironment (TME) interferes with anti‐tumoral immune responses.^8^, ^9^ As such, understanding and targeting the TME can reveal avenues for developing efficient immunotherapies against numerous tumors. Several methods such as gene set enrichment analysis (ssGSEA), Microenvironment Cell Populations (MCP)‐counter, CIBERSORT, and Xcell have been developed to analyze the infiltration of immune cells in cancer tissues.^10^, ^11^, ^12^, ^13^ Most of the T cell infiltrations (80%) occur in metastatic cancers.^14^ Meanwhile, the nature and extent of immune cell infiltration into solid tumors have been reported to influence therapeutic responses. Based on the infiltration of cytotoxic immune cells in the TME, cancers can be classified into immunologically active “inflamed” (hot) tumors and inactive “non‐inflamed” tumors (cold) tumors. Cold tumors are insensitive to either chemotherapy or immunotherapy, thus are associated with poor prognosis. Hot tumors are characterized by the infiltration of leukocytes including CD8^+^ T cells.^15^ Other important tumor‐related factors include CCL5, CXCL9, and CXCL10, which regulate the recruitment of T cells into tumors.^16^ In contrast, even though cold tumors display are infiltrated by macrophages, the infiltration level of CD8^+^ T cells and antigen‐presenting factors such as HLA class I molecules is reduced in these tumors.^17^ Cancer resists immune response by upregulating the expression of immune checkpoint components on their cell surfaces, which inhibits T cell response in the TME.

In this study, osteosarcomas were divided into cold and hot tumors based on the immune infiltration score. The infiltration level of immune cells across tumor types was analyzed using ssGSEA, MCP‐counter, CIBERSORT, and Xcell. A prognosis predictive model for osteosarcoma was constructed and validated in patients receiving immunotherapy. The results showed that the infiltration of B and T cells in men with osteosarcoma was higher relative to that in women. In addition, differently expressed genes (DEGs) between cold and hot tumors were identified. Notably, NPTX2 was over‐expressed in cold tumors, which negatively correlated with the expression of CD8A, GZMB, and IFNG. Knockdown of NPTX2 increased CCL4 secretion. In vitro functional analysis revealed that the inhibition of NPTX2 in HOS and SW1353 cells reduced cell proliferation and promoted cell apoptosis. Overall, the constructed model accurately predicted the prognosis of osteosarcoma patients undergoing immunotherapy.

## 2 MATERIALS AND METHODS

### 2.1 Source of data

**FIGURE 1:**
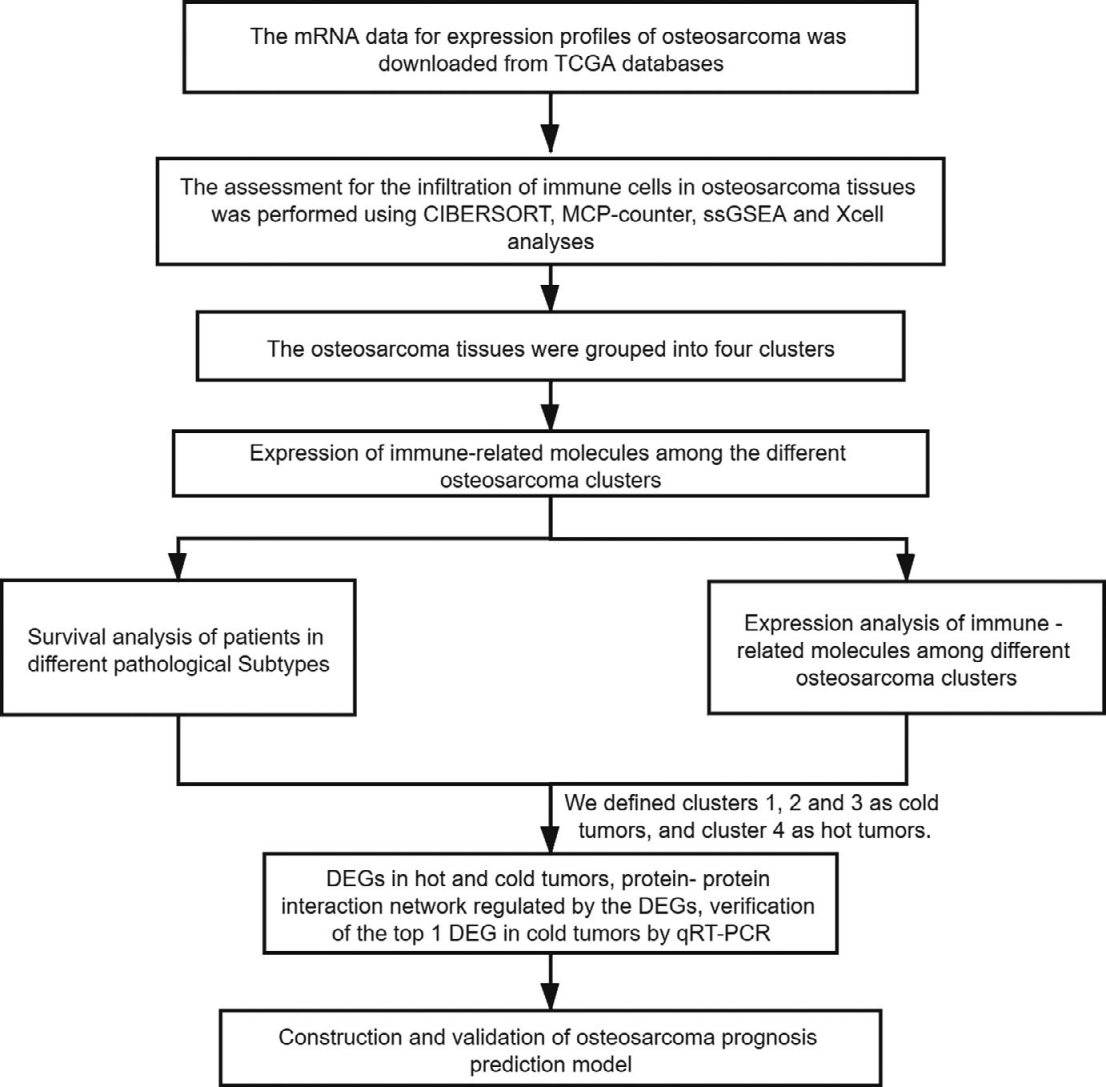
The general research design and flow of the study

The mRNA sequencing and clinical data of 119 osteosarcoma patients were downloaded from the TCGA portal (http://tcga.cancer.gov/dataportal).^18^ The study design and features of samples used at each stage of the analysis are shown in Figure 1.

### 2.2 Infiltration of immune cells

Infiltration of immune cells to tumor sites was assessed using the ssGSEA using R software.^19^ Infiltration rate of 22 immune cells was analyzed using CIBERSORT (http://cibersort.stanford.edu/). The population and proportion of tumor microenvironment cells were analyzed using the “MCP‐Counter” package in R software.^12^ The Immune scores (ISs), Stromal Scores (SSs), and Tumor Purity (TP) were calculated using the “ESTIMATE” package in R software. The inclusion criteria were: *p* < 0.05. The “ggplot2” package was used to draw violin diagrams to visualize differences in the infiltration of immune cells.

### 2.3 Cell consensus clustering

Consensus clustering provides quantitative and visual estimates of unsupervised classes in a dataset.^20^ Classification of osteosarcoma patients into various clinically significant subtypes was performed using the “ConsensusClusterPlus” package (http://www.bioconductor.org/). The clusters were visualized using a heat map and delta diagram.

### 2.4 Expression of immune‐related molecules across different osteosarcoma clusters

The TP, ISs, and SSs of each osteosarcoma cluster were calculated using the ESTIMATE algorithm in the packager software. The expression and function of immune‐related molecules such as antigen‐presenting proteins, chemokine, cytokines, and immune checkpoints were analyzed and plotted using the “ggplot2” package.

### 2.5 Pathological subtypes and survival analysis of different osteosarcoma subgroups

Overall survival (OS) and physiological vulnerability index (PFI) between different subgroups were also determined. PFI reflects the accumulation of age‐related defects.^21^ Survival analyses were performed using the “survival” package in R software, whereas corresponding box plots were constructed using the “ggplot2” package. Immune scores for each cluster were calculated and compared. Tumors in each cluster were further classified into hot and cold tumors. Survival analyses of patients with hot or cold tumors were also performed. Data were normalized using the “Sva” package.^22^ The TME of cold and hot tumors was analyzed using the limma package. Genes were considered up‐regulated based on |Log2FC| >1, and downregulated based on |Log2FC| >1, adjusted *p*‐value <0.05.^23^

### 2.6 Identification of differently expressed genes between cold and hot tumors

Fresh osteosarcoma tissues collected after surgery were washed three times using PBS before treatment with TRIzol (Invitrogen) for RNA extraction. The RNA was reverse transcribed to cDNA and subjected to quantitative PCR using the Rever Tra Ace qPCR RT Kit and SYBR Premix Ex Taq following the manufacturer's instructions. The GAPDH mRNA served as the internal control. The primers used were as follows: Human Neuronal pentraxin Ⅱ (NPTX2); F: ACGGGCAAGGACACTATGG; R: ATTGGACACGTTTGCTCTGAG, Human CD8A; F: TCCTCCTATACCTCTCCCAAAAC, R: GGAAGACCGGCACGAAGTG, Human IFNG; F: TCGGTAACTGACTTGAATGTCCA, R: TCGCTTCCCTGTTTTAGCTGC, Human GZMB; F: TACCATTGAGTTGTGCGTGGG, R: GCCATTGTTTCGTCCATAGGAGA, Human GAPDH: F: GAGTCAACGGATTTGGTCGT, R: TTGATTTTGGAGGGATCTCG. The association of NPTX2 with CD8A, GZMB, and IFNG expression was analyzed using Spearman correlation. The correlation analyses were performed using GraphPad Prism (version 7.00).

### 2.7 Cell culture

Human osteosarcoma cancer cell lines (HOS, SW1353) were purchased from the China Center for Type Culture Collection (CCTCC). The HOS cell lines were cultured in MEM supplemented with 10% heat‐inactivated fetal bovine serum (FBS), 100 U/ml of penicillin, and 100 mg/ml of streptomycin. The SW1353 cell lines were maintained in L‐15 enriched with 10% FBS, 100 U/ml of penicillin, and 100 mg/ml of streptomycin. All cultures were incubated in a humidified atmosphere of 5% CO_2_ at 37℃.

### 2.8 siRNA‑mediated gene knockdown

siRNA targeting NPTX2 (5′‐GCGCACAAGAAAUUGUCAATT‐3′ (sense) and 5′‐UUGACAAUUUCUUGUGCGCTT‐3′ (antisense) for *NPTX2*‐siRNA1, 5′‐GGUGGACAAUAACGUCGAUTT‐3′ (sense), and 5′‐AUCGACGUUAUUGUCCACCTT‐3′ (antisense) for *NPTX2*‐siRNA2. 5′‐CUCCGCACAAACUACCUAUTT‐3′ (sense) and 5′‐AUAGGUAGUUUGUGCGGAGTT‐3′ (antisense) for *NPTX2*‐siRNA3) and scrambled negative control siRNA were provided by Shanghai GenePharma. Transfection siRNAs into HOS and SW1353 cells were performed using Lipofectamine 2000 (Invitrogen) at a final concentration of 80 nM. The efficiency of siRNA knockdown was subsequently confirmed using qPCR (as aforementioned).

### 2.9 Cell proliferation assay

Cell Counting Kit‐8 (DOJINDO) was used according to the manufacturer's instructions. Briefly, transfected osteosarcoma cells were seeded in 96‐well plates at a density of 5000 cells per well and incubated for 24, 48, and 72 h. Next, 10 μl of CCK‐8 reagent was added into each well every 24 h and incubated at 37℃ for 2 h, after which the absorbance was measured at 450 nm (ELx800, Bio‐Tek).

### 2.10 Apoptosis assay

Seventy‐two hours after transfection, cells were harvested, washed, and suspended in Annexin V binding buffer (BioLegend). Next, they were treated with Annexin V Alexa Fluor 647 (BioLegend, Inc.) for 15 min at 4℃ in the dark. The propidium iodide (PI; Sigma‐Aldrich; Merck Millipore) was added to the cells and analyzed by flow cytometry immediately.

### 2.11 ELISA

Levels of CCL4 and CXCL13 in supernatants of transfected osteosarcoma cells were measured using the ELISA kit (Elabscience) according to the manufacturer's instructions. Absorbance was measured at 450 nm with a microplate reader (ELx800, Bio‐Tek).

### 2.12 Signaling and metabolic pathways associated with the DEGs

Gene ontology (GO) terms and Kyoto Encyclopedia of Genes and Genomes (KEGG) enrichment analysis were performed using the “Clusterprofiler” package in R software.^24^ The protein–protein interaction (PPI) network related to the DEGs was constructed using STRING (https://string‐db.org/). The significance of interaction scores was set at 0.9.^25^ Hub genes were selected based on *p* < 0.05 and q < 0.05. The analysis was performed using in Cytoscape software.

### 2.13 Construction of prediction model

A prognostic prediction model (adj *p* < 0.05) was constructed using the DEGs between cold and hot tumors. Multivariate models of immune‐related genes were constructed using the glmnet” package based on the most minor absolute shrinkage and selection operator (LASSO) Cox regression method.^26^, ^27^ The top most DEGs were selected based on lasso penalty Cox regression analysis. The prediction performance of the DEGs was assessed using the LASSO regression model. The independence of the prognostic model was analyzed using multivariate cox regression analysis. The accuracy of the gene model was assessed based on the area under the ROC curve (AUC).

### 2.14 Validation of the predictive model

The performance of the prediction model composed of 11 hub genes was validated in an external gene expression dataset of 429 osteosarcoma patients.^28^ Patients were divided into high and low‐risk groups based on the expression score of the 11 hub genes. Differences in the OS of patients in the two groups of patients were analyzed using the R package.

### 2.15 Statistical analysis

All analyses were performed using R version 3.6.3. Differences in the infiltration of immune cells between hot and cold tumors were analyzed using Wilcox Test, whereas differences in immune scores, transformation scores, and tumor purity among the four osteosarcoma clusters were analyzed using ANOVA. Statistical significance was set at *p* < 0.05.

## 3 RESULTS

### 3.1 Infiltration of immune cells in osteosarcoma

**FIGURE 2:**
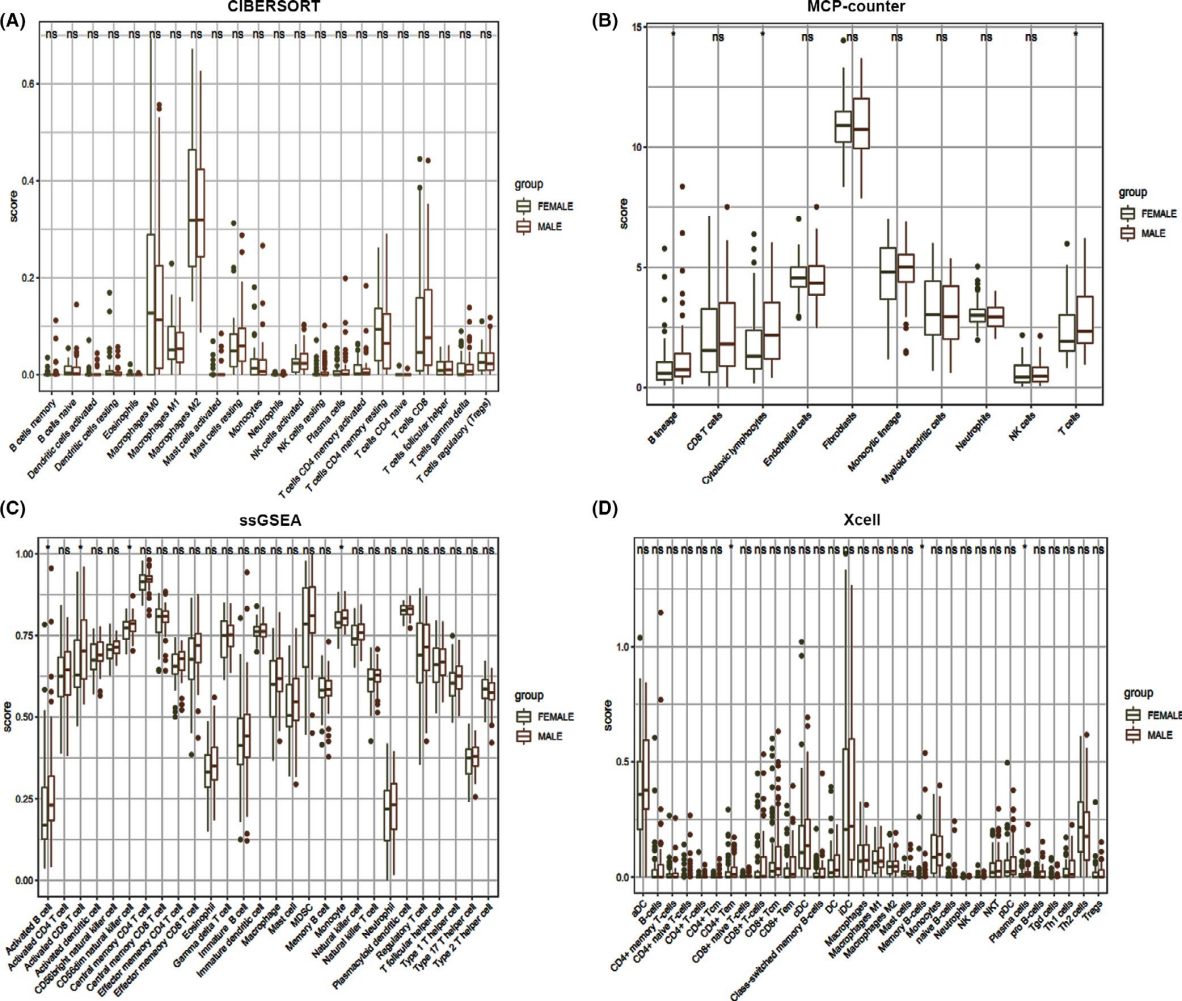
The infiltration profile of different immune cells in TME for men and women with osteosarcoma. The analysis was performed using (A) CIBERSORT, (B) MCP‐counter, (C) ssGSEA, and (D) Xcell computational method

Analysis using CIBERSORT, MCP‐counter, ssGSEA, and Xcell showed higher infiltration of Memory‐B cells, activated B cells, and activated CD8^+^ T cells in TME in male osteosarcoma patients compared with female patients. Monocytes and CD56^+^ dim natural killer (NK) cells were more abundant in osteosarcoma tissues of male patients showing the importance of innate immunity in osteosarcoma. This finding partially explains the difference in efficacy of immunotherapy between male and female osteosarcoma patients (Figure 2A–D). The synergy of multiple cells results in immune infiltration in the tumor microenvironment. Further analyses showed significant correlations between infiltration levels of several immune cells. These associations are presented in Figure S1A–D. Notably, a significant positive correlation was observed between infiltration of activated CD8^+^ T cells and activated B cells as well as effector memory CD8^+^ T cells, implying that these cells exhibit a synergistic anti‐tumor effect. In addition, myeloid‐derived suppressor cells (MDSC) expression was positively correlated with regulatory T cells (Tregs), activated CD8^+^ T cells, effector memory CD8^+^ T cells, type 1 T helper cell, and activated dendritic cells. This is probably because the immunosuppressive tumor activities are only induced upon the activation of the immune system.

### 3.2 Clustering of osteosarcoma patients based on the immune infiltration profile

**FIGURE 3:**
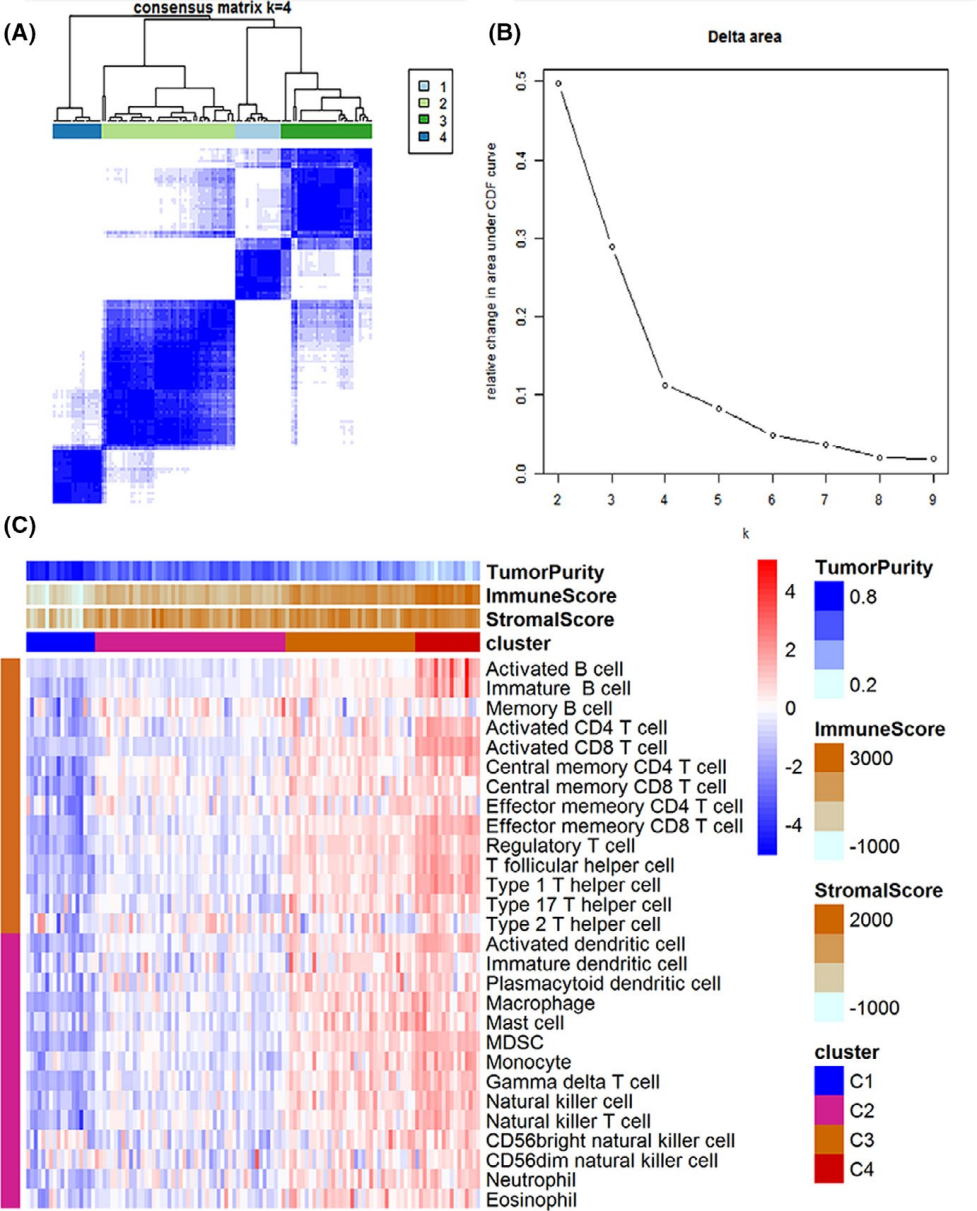
Consistent clustering diagram of immune cells in different clusters. A heatmap showing the distribution of immune genes (A), and four subgroups of CDF (B). (C) A heatmap showing the infiltration level of immune cells in the four osteosarcoma subgroups. The clusters were generated based on TP, ISs, and SSs

Osteosarcoma patients were grouped into further immune infiltration clusters based on ssGSEA results. Cumulative distribution function (CDF) plots for the infiltration of immune cells were generated (Figure 3A and B). Four subgroups were identified. A heat map was generated to show the distribution of various immune cells in osteosarcoma tissues of patients in the four groups (Figure 3C). Further analyses were performed to explore differences between innate and adaptive immunity, TP, and SSs. The findings showed a gradual increase in infiltration of tumor cells in tumor tissues from clusters 1 to 4. Tumor purity was lowest in tissues in cluster 4 compared with the other clusters. Notably, cluster 4 tissues exhibited the highest ISs and SSs.

### 3.3 Immune score and expression of immune‐related genes between clusters

**FIGURE 4:**
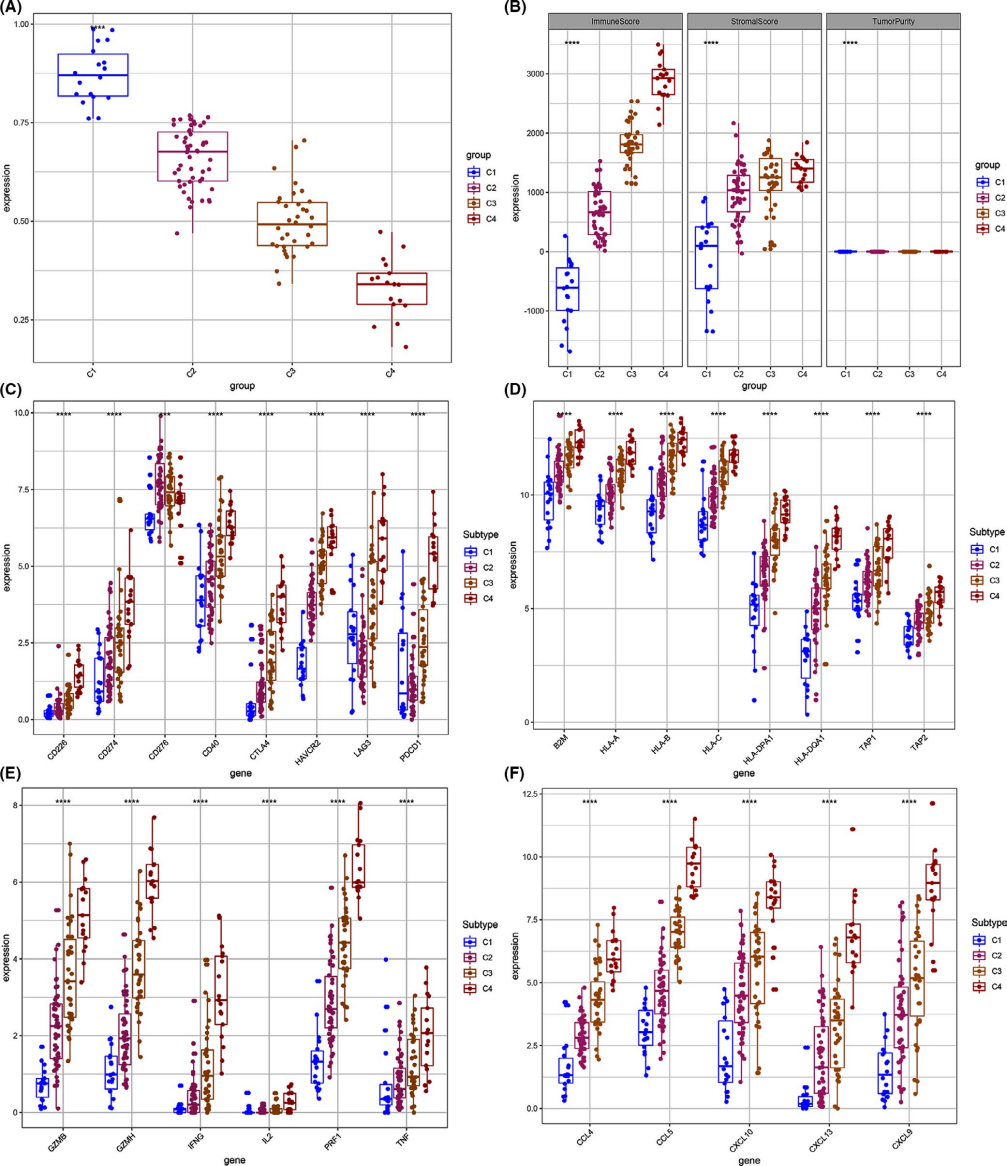
Box plot displaying the expression pattern of immune‐related genes between osteosarcoma subgroups. (A and B) ISs and SSs for the four osteosarcoma clusters. Compared with the other three clusters, cluster 4 had a higher immune score. (C–F) The expression levels of multiple immune genes among the four clusters. *, *p* < 0.05. **, *p* < 0.01. ***, *p* < 0.001. ****, *p* < 0.0001

TP, ISs, and SSs of the four osteosarcoma sub‐groups were determined based on immune cell infiltration (Figure 4A and B). Cluster 4 showed the highest ISs and SSs. These findings indicate that ISs and SSs can be used to reflect the cancer subtype. Box plots showing the relationship between expression of immune checkpoints (CD226, CD274, CD276, CD40, CTLA4, HAVCR2, LAG3, PDCD1), common antigen‐presenting molecules (B2 M, HLA‐A, HLA‐B, HLA‐C, HLA‐DPA1, HLA‐DQA1, TAP1, TAP2), cytokines (GZMB, GZMH, IFNG, IL2, PRF1, TNF), and chemokines (CCL4, CCL5, CXCL10, CXCL13, CXCL9) were generated (Figure 4C–F). All these molecules were over‐expressed in cluster 4 tissues compared with the other clusters. Therefore, specific immune infiltrates in cluster 4 tissues were further analyzed and compared with those of the other clusters. Cluster 4 tissues showed low expression levels of CD276 compared with the expression levels in tissues of clusters 2 and 3. This finding implies that CD276 is a potential therapeutic target for osteosarcoma immunotherapy (Figure 4C).

### 3.4 Survival analysis among pathological subgroups

**FIGURE 5:**
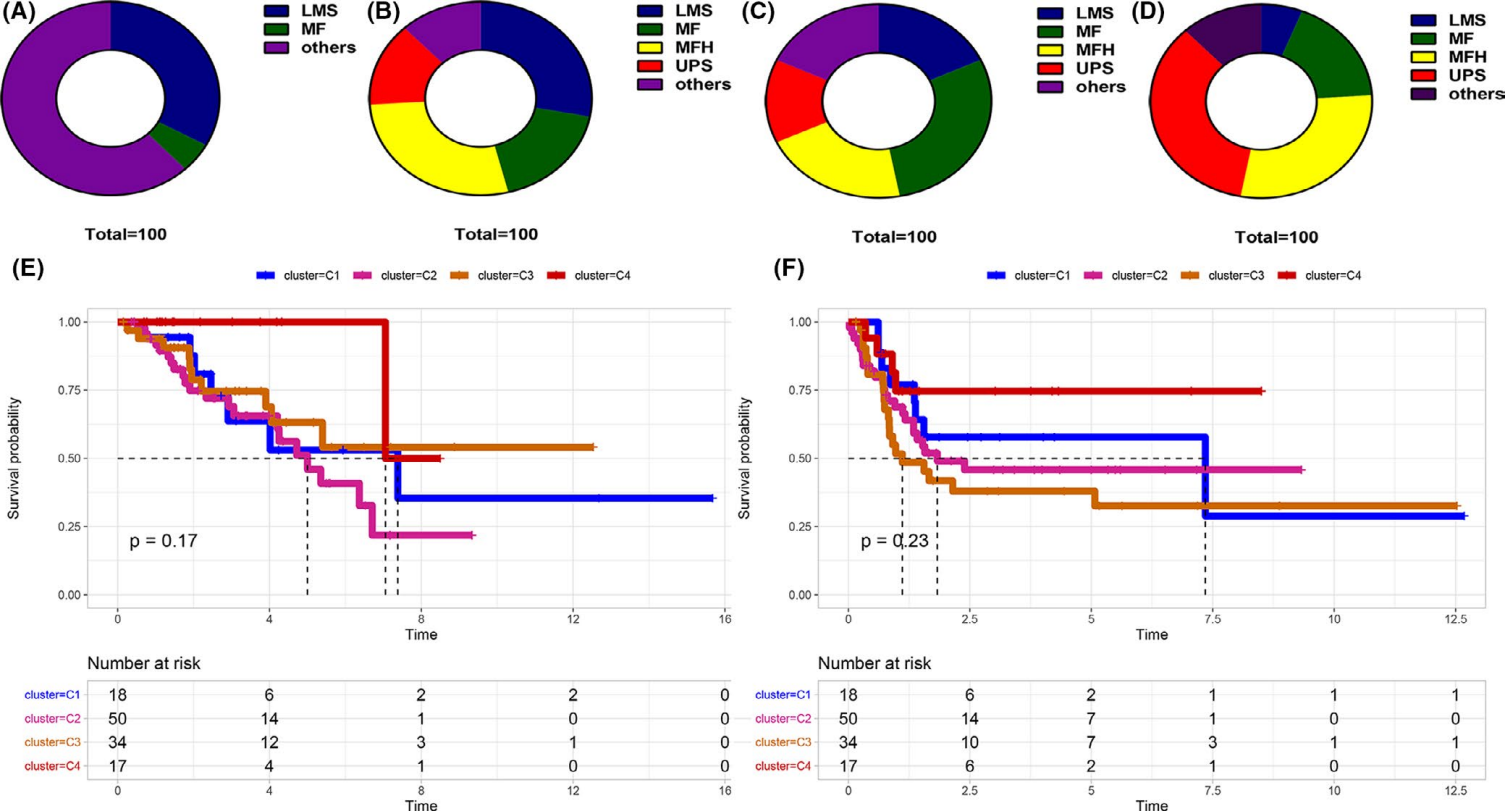
Distribution of the main pathological osteosarcoma subtypes in the four osteosarcoma clusters. (A–D) The major pathological subtypes including Leiomyosarcoma (LMS), Myxofibrosarcoma (MF), undifferentiated pleomorphic sarcoma (MFH), and Undifferentiated Pleomorphic Sarcoma (UPS) in the four clusters. (E and F) The survival rate of patients in different subtypes

Osteosarcoma exhibits several pathological subtypes including leiomyosarcoma (LMS), myxofibrosarcoma (MF), undifferentiated pleomorphic sarcoma (MFH), and undifferentiated pleomorphic sarcoma (UPS). The distribution of each pathological subtype in the four subgroups was explored (Figure 5A–D). Cluster 1 mainly comprises the non‐major pathological subtypes. Cluster 4 mainly comprised UPS and MFHs compared with the other clusters. Furthermore, patients in cluster 4 exhibited longer OS (*p* = 0.17, *p* = 0.23) compared with those in clusters 1–3 (Figure 5E and F).

### 3.5 Survival analysis of patients with cold and hot tumors

**FIGURE 6:**
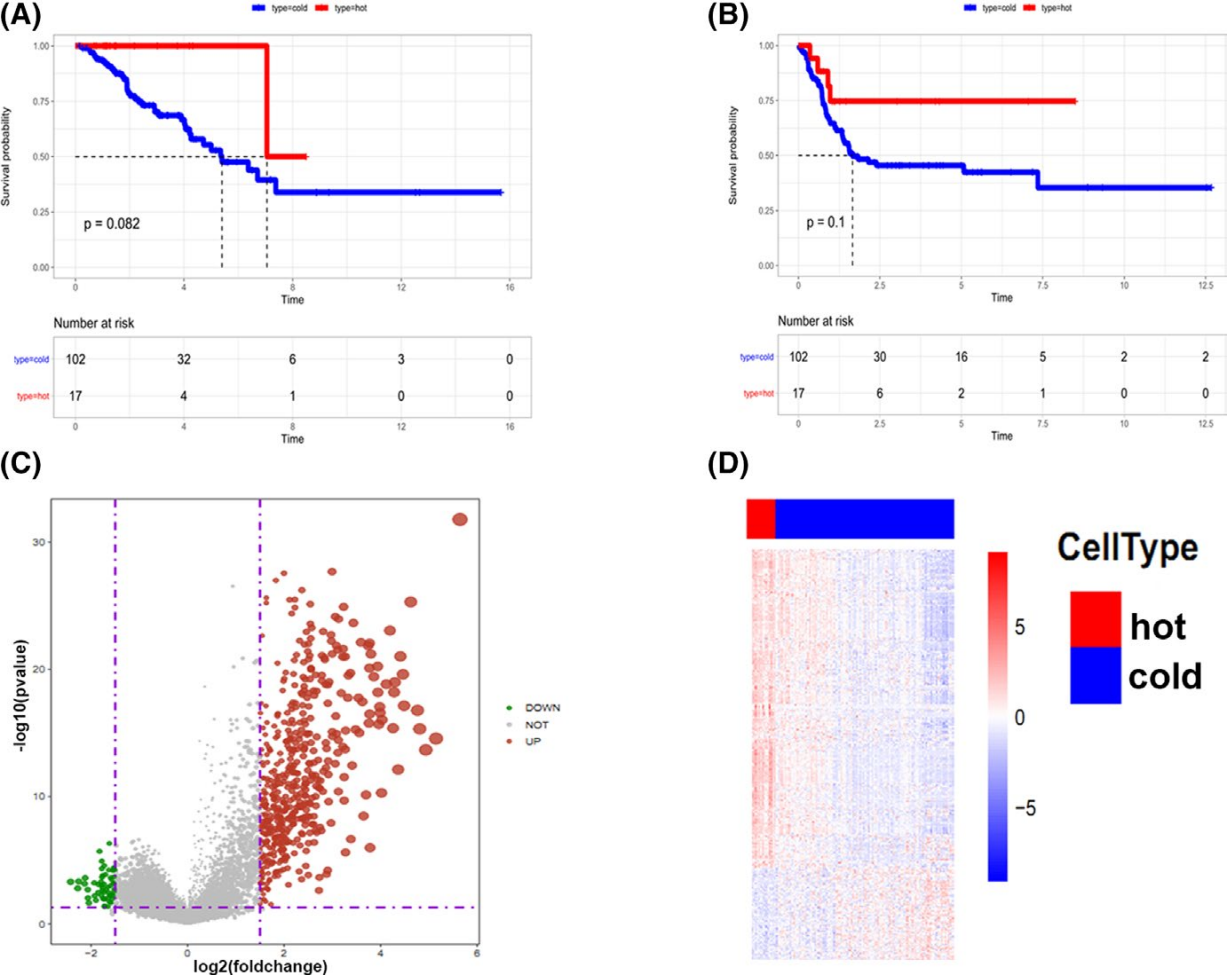
Survival rate of patients with cold and hot tumors. (A and B) Survival rate of hot and cold tumors. (C) A heat map and (D) volcano map of the DEGs between hot and cold tumors. The grey line represents normal gene expression, green line represents down‐regulated expression whereas red line represents up‐regulated genes, both for heat and volcano plots

Cluster 4 tissues showed higher immune score and immune gene expression compared with the other clusters. In addition, patients in cluster 4 displayed better prognosis. For further analysis, cluster 4 was defined as hot tumors, whereas the others (cluster1‐3) were defined as cold tumors. Analyses showed that hot tumors were associated with favorable clinical outcome (Figure 6A and B); however, the difference was insignificant. This can be attributed to the few samples used for the hot tumors. The difference in gene expression between cold and hot tumors was presented as volcano and heat maps (Figure 6C and D). Analysis showed a total of 818 differentially upregulated and 1054 differentially downregulated genes between hot and cold tumors. The top 6 most upregulated genes in the cold tumor tissues are shown in Table 1.

**TABLE 1:** The top 6 up‐regulated genes in cold tumors

Gene symbol	|Log FC|	*p* value	Function
NPTX2	2.42	0.00049	Involved in excitatory synapse formation
CA9	2.28	0.00157	Involved in various biological processes
RASL11B	2.26	0.00045	Includes GTP binding and ferrous iron transmembrane transporter activity
ACAN	2.13	0.00069	Includes calcium ion binding and extracellular matrix structural constituent
SEZ6L2	2.12	0.00024	Contributes to specialized endoplasmic reticulum functions in neurons
PTN	2.07	0.00200	Involved in cell growth, cell migration, angiogenesis, and tumorigenesis

### 3.6 NPTX2‐augmented immunosuppression in the osteosarcoma tumor microenvironment

**FIGURE 7:**
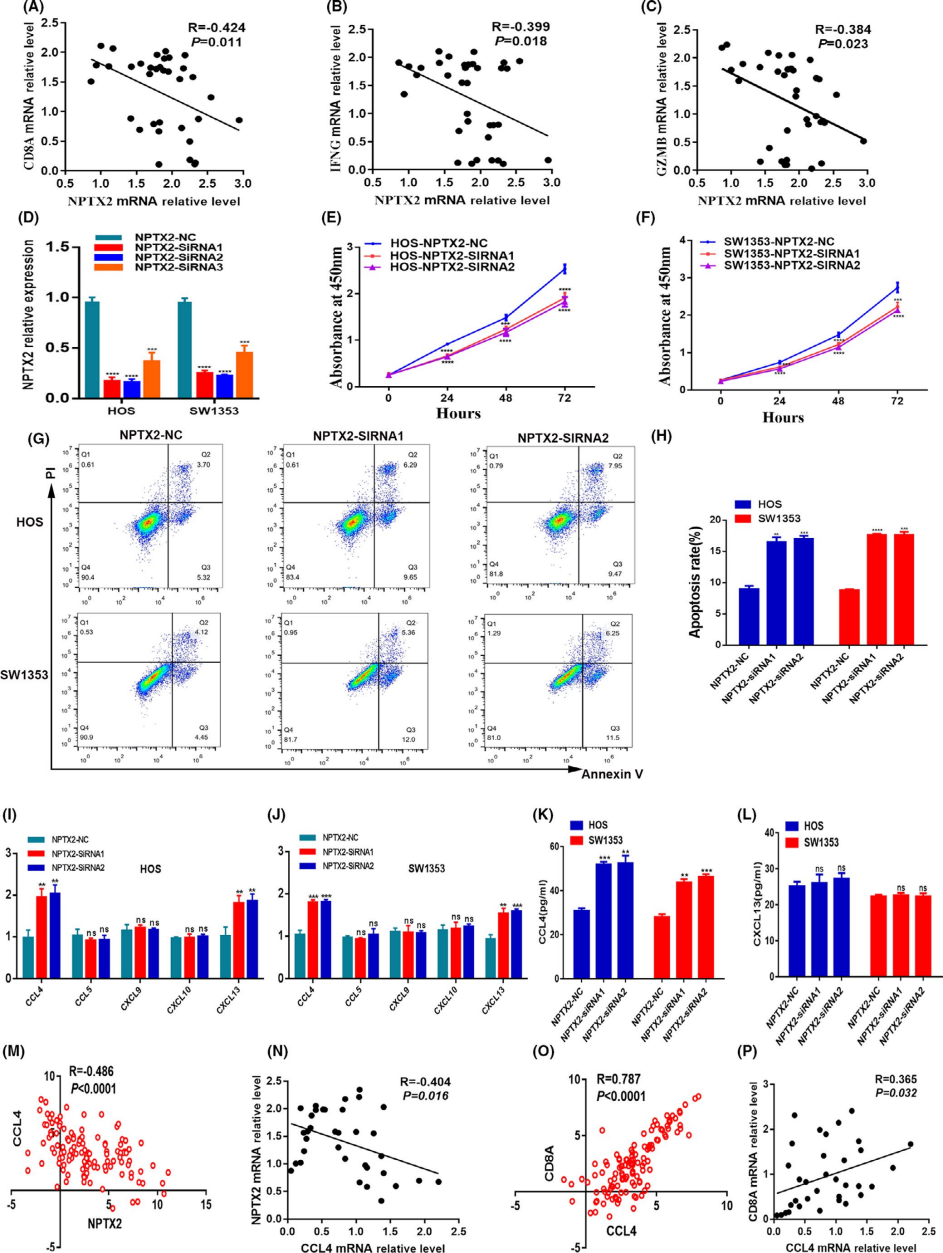
Validation of NPTX2 function in osteosarcoma. (A–C) Association of NPTX2 mRNA with CD8A, IFNG, and GZMB expression in osteosarcoma tissues (*n* = 35). (D) The knockdown efficacy of NPTX2 in HOS and SW1353 cells as determined by qPCR. (E and F) Cell proliferation was measured with the CCK8 assays in HOS and SW1353 cells transfected with siRNAs or NC. (G and H) Apoptosis of HOS cells and SW1353 cells after transfection with siRNAs or NC was detected by flow cytometric assay. (I and J) The expression of CCL4, CCL5, CXCL9, CXCL10, and CXCL13 as measured with qPCR in HOS‐NPTX2‐NC and HOS‐NPTX2‐siRNAs cells, or SW1353‐NPTX2‐NC and SW1353‐NPTX2‐siRNAs cells. (K and L) The expression of CCL4, and CXCL13 as quantified with ELISA assay. (M and N) Association of NPTX2 mRNA with CCL4 expression in the TCGA datasets and in osteosarcoma tissues from our patients (*n* = 35). (O and P) Association of CCL4 mRNA with CD8A expression in the TCGA datasets and osteosarcoma tissues from our patients (*n* = 35)

NPTX2 was the most dysregulated gene, therefore, subsequent experiments focused on several aspects of this gene. Analysis showed that the expression of NPTX2 genes was negatively correlated with the expression of CD8A and cellular immune killer molecules (GZMB, IFNG) (Figure 7A–C). To further explore the role of NPTX2 in osteosarcoma, siRNA was used to knockdown NPTX2 in osteosarcoma cell lines, HOS, and SW1353. Knockdown efficiency was confirmed by qPCR analysis (Figure 7D). Analysis was performed to explore if NPTX2 affects the proliferation and apoptosis of osteosarcoma cells. CCK8 assay showed that the knockdown of NPTX2 inhibited the growth of HOS and SW1353 cells (Figure 7E and F). In addition, NPTX2 knockdown significantly increased the apoptosis rate in osteosarcoma cells (Figure 7G and H). To explore the mechanisms of NPTX2 underlying the inhibition of CD8^+^ T cells recruitment, the expression levels of chemokines that could potentially recruit T cells, including CCL4, CCL5, CXCL9, CXCL10, and CXCL13 in NPTX2‐siRNA and control cells were determined. The results showed an increase in expression levels of CCL4 and CXCL13 in NPTX2‐siRNA cells compared with the levels in the control group (Figure 7I and J). Further, the level of CCL4 and CXCL13 was explored using ELISA. The results showed that the level of CCL4 was higher in NPTX2‐siRNA cells, indicating that NPTX2 inhibits CCL4 production (Figure 7K and L). Notably, the local expression of CCL4 in tumor lesions was negatively correlated with the expression of NPTX2 in the TCGA datasets and in osteosarcoma patient samples (Figure 7M and N). In addition, a positive correlation was observed between the expression level of CCL4 and expression level of CD8A (Figure 7O and P). These findings indicate that NPTX2 inhibits CCL4 secretion in osteosarcoma cells, which can further affect the recruitment of CD8^+^ T cells into the tumor microenvironment. This finding implies that NPTX2 promotes osteosarcoma development by suppressing anti‐tumor responses.

### 3.7 Protein interaction and pathways associated with DEGs

**FIGURE 8:**
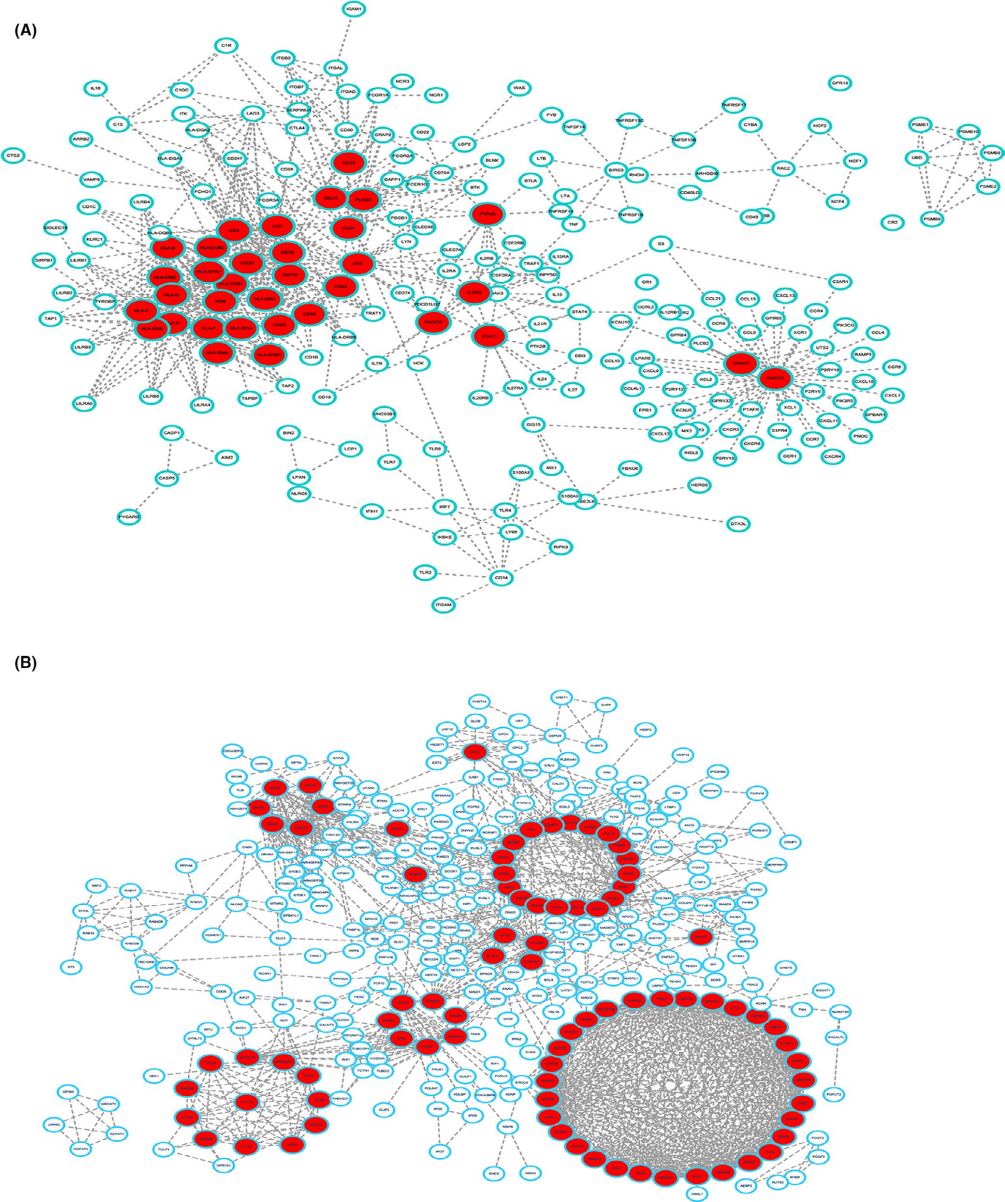
A PPI network of the DEGs between cold and hot tumors. (A) Down‐regulated and (B) up‐regulated DEGs between cold and hot tumors. (PPI, Protein–Protein Interaction; DEGs, Differentially expressed genes)

GO analyses showed that downregulated DEGs between cold and hot tumors were mainly involved in the regulation of biological process (BP) and expression of cellular components (CC) such as morphogenesis, neural differentiation, and development of adherens junction. Moreover, upregulated DEGs were mainly implicated in the regulation of immune responses such as T cell activation and regulation of leukocytes (Figure S2A–D). These findings indicate that over‐expressed genes are involved in the regulation of inflammatory responses against tumors, mainly T cell responses. KEGG analysis showed that up‐regulated DEGs are implicated in cytokine−cytokine receptor interaction, viral protein–cytokine interaction, and cytokine receptor and hematopoietic signaling pathways (Figure S2E–H). This finding shows that upregulated genes play important roles in immune response in hot tumors. A PPI network of proteins regulated by DEGs in cold and hot tumors was constructed (Figure 8A and B). The network shows protein interactions regulated by 34 upregulated and 83 downregulated DEGs, respectively. The PPI network showed extensive interactions between stromal signature and immune signature of DEGs, which may be related to the high immune infiltration of hot tumors.

### 3.8 Prognosis predictive value of the hub genes

**FIGURE 9:**
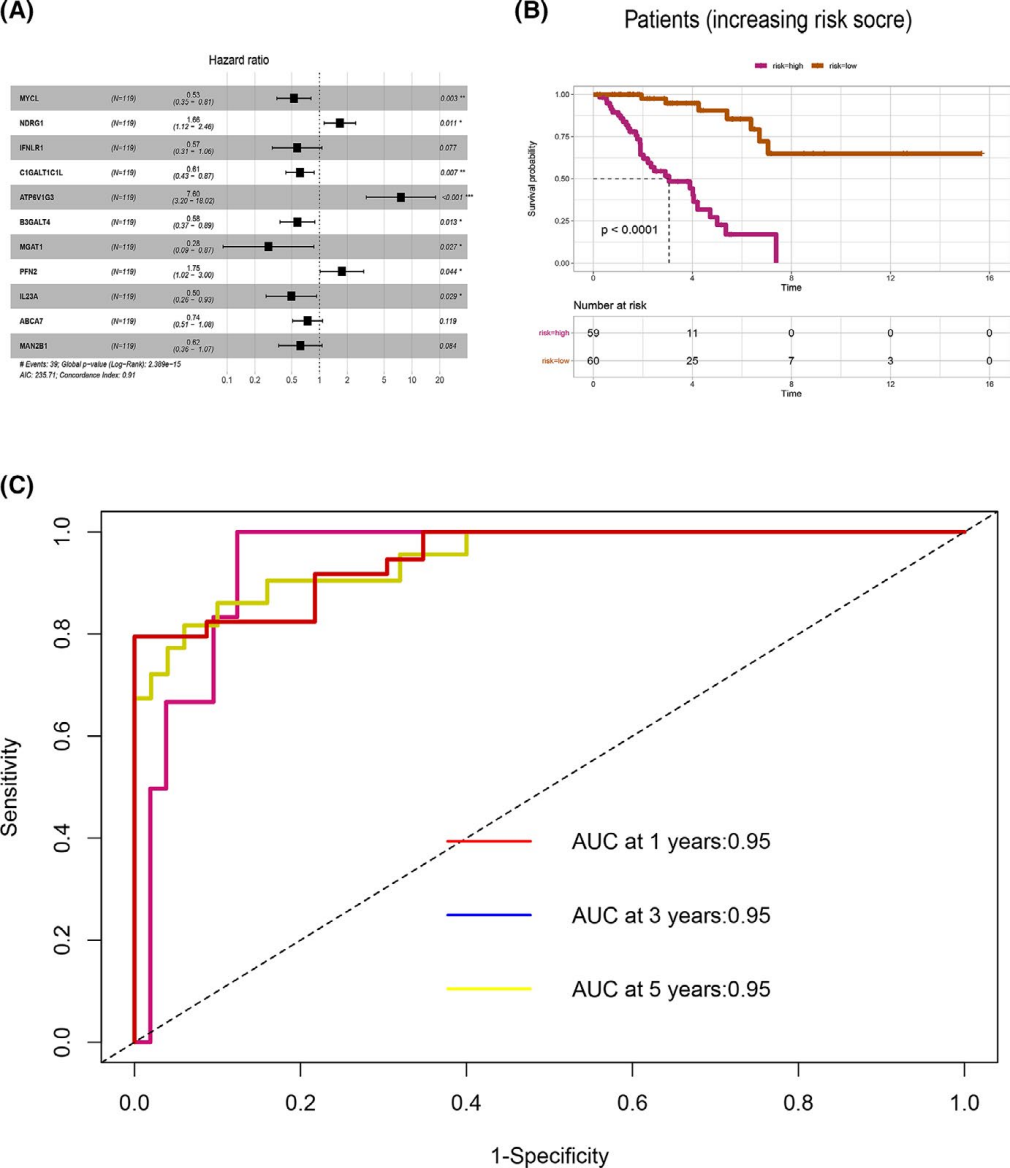
Hub genes for the osteosarcoma prognostic model. (A) Multivariate cox regression analysis of hub genes. (B) Kaplan–Meier curves showing the OS of patients with high and low prognostic scores. (C) The time‐dependent ROC curves displaying the prognostic prediction of the hub genes model

LASSO analysis of 34 most upregulated and 83 downregulated DEGs was performed before deriving the predictive model (Figure S3A). LASSO analysis showed 11 hub genes highly correlated with OS of osteosarcoma patients. The risk score for osteosarcoma patients and its relationship with OS is shown in Figure S2B and C. Prognostic value of the hub genes was then evaluated. Osteosarcoma patients were grouped into high and low‐risk groups based on the risk scores generated using the model. The survival curve of the two groups of patients based on risk scores is shown in Figure 9B. A low score was correlated with better OS (*p* < 0.001). The accuracy of the model for the prognosis of osteosarcoma is presented in Figure 9A. The area under ROC curve (AUC) for the 1, 3, and 5‐year OS of osteosarcoma patients was 0.95 (Figure 9C), implying that the model was accurate in predicting OS.

### 3.9 Validation of the predictive value of the model

**FIGURE 10:**
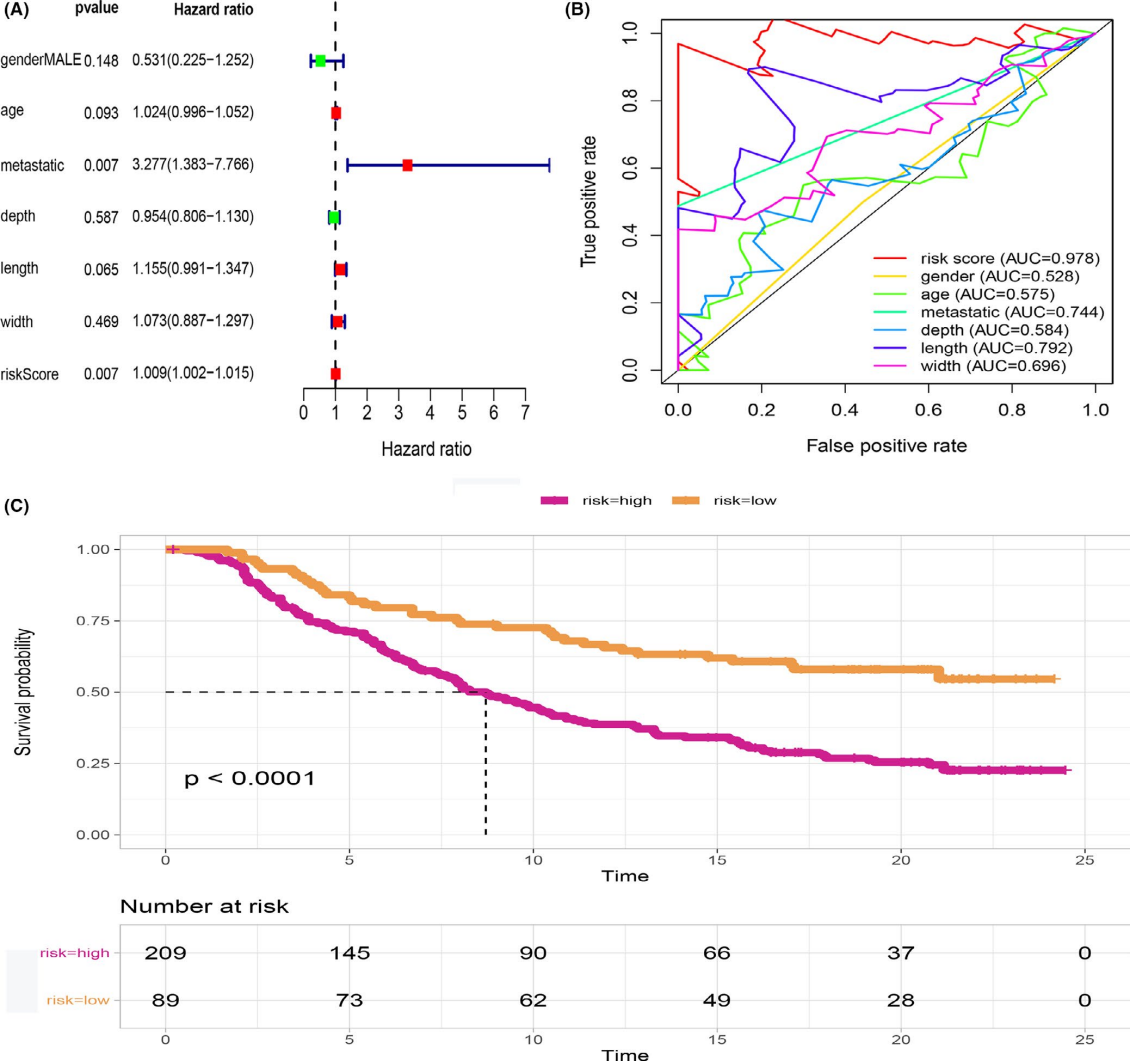
Relationship between clinical features and prognosis of osteosarcoma. (A) Multivariate cox regression analysis results indicating the relationship between metastasis, risk score, and OS. (B) The ROC showing the prognostic accuracy of the 11‐gene model across several cancer and disease factors (gender, across age, in metastatic, and non‐metastatic tumors as well as depth, length, and width). (C) The predictive accuracy of the 11‐gene risk score model in predicting immunotherapy response. The low‐risk group had better OS than the high‐risk group (*p* < 0.0001)

Risk scores for osteosarcomas were calculated using the 11 selected hub genes. Multivariate COX regression analysis was performed for the effect of patient and disease factors on the overall survival (OS) of individuals with osteosarcoma (Figure 10A). The factors in multivariate COX regression analysis included gender, age, tumor metastasis, depth, length, width, and risk Score. The findings showed that metastasis (*p* = 0.007) and risk score (*p* = 0.007) were significantly correlated with OS.AUC for sensitivity and specificity of the model are shown in Figure 10B. AUCs for gender, age, metastatic, depth, length, width, and risk score were 0.528, 0.575, 0.744, 0.584, 0.792, and 0.978, respectively. The efficacy of the 11‐gene model was validated using external clinical and RNA‐seq data of 298 patients with metastatic urothelial cancer who received anti‐PD‐L1 therapy (atezolizumab).^28^ Patients were divided into high and low‐risk groups based on the risk scores of the 11 hub genes before survival analyses (Figure 10C). Analysis showed that low‐risk group patients exhibited better OS compared with the high‐risk group patients (*p* < 0.0001). These findings show that the 11‐gene risk Score model is accurate in predicting the prognosis of osteosarcoma patients.

## 4 DISCUSSION

Osteosarcoma is the most prevalent primary bone cancer with a detrimental impact on the life of several people. Although surgery and adjuvant chemotherapy are the mainstream treatment methods for cancer, immunotherapy has become a new treatment modality for numerous solid tumors. However, immunotherapy against osteosarcoma is yet to be developed. Herein, we analyzed RNA‐sequences of osteosarcoma tissues in the TCGA database and revealed the hub genes associated with infiltration of immune cells in different types of osteosarcoma. The findings provide a theoretical foundation for the development of immunotherapy for osteosarcoma.

Although immunotherapy has revolutionized cancer treatment, this approach is only infective in patients with high infiltration of immune cells and molecules in TME. Compelling evidence indicates that tumor‐infiltrating lymphocytes (TILs) in TME impact the efficacy of immunotherapy ^29^ for glioblastoma multiforme,^30^ breast cancer,^31^ and lung cancer.^32^ Cancers can be classified as cold and hot tumors according to immune TME. Particularly, hot or inflammatory tumors are characterized by high infiltration of leukocytes, including CD8^+^ T cells.^15^

Moreover, a previous study found that the efficacy of cancer immunotherapies differs between men and women,^33^, ^34^ which concur with our findings. Male osteosarcoma patients display higher infiltration of memory and activated B cells, and activated CD8^+^ T cells relative to female counterparts. Besides, men have more monocytes and CD56^+^ bright natural killer (NK) cells than women, which mediates innate response against osteosarcoma. This potentially justifies the difference in osteosarcoma immune response between men and women. Herein, we revealed that the expression of activated CD8^+^ T cells in osteosarcoma patients was positively correlated with that of activated B cells and memory CD8^+^ T cells. The anti‐tumor effect of B cells in osteosarcoma is elusive; however, B cells influence ICB treatment in melanoma through the alteration of the activation and functioning of CD8^+^ T cells.^35^

In this study, osteosarcoma patients were classified into several clusters based on immune infiltration. Patients with greater immune response exhibited better OS. Cluster 4 tumors demonstrated similar properties as hot tumors, whereas the characteristics of clusters 1–3 tumors were comparable to those of cold tumors. NPTX2 expression correlated with that of CD8A. NPTX2 is a member of the neuronal pentraxin family ^36^ and is strongly expressed in tissues of numerous cancer types, including malignant gliomas, lung cancer, and pancreatic cancer.^37^, ^38^, ^39^ However, NPTX2 expression in osteosarcoma has not been reported. We found that NPTX2 was the most dysregulated protein in cold tumors. Also, NPTX2 expression was negatively correlated with CD8A, GZMB, and IFNG. Emerging evidence suggests that CCL4 potentially drives the recruitment of CD8^+^ T cells.^40^, ^41^, ^42^ Additionally, we found that the level of CCL4 was elevated via siRNA‐induced NPTX2 silencing. In tumor lesions, CCL4 expression was negatively correlated with the expression of the NPTX2, which implied that NPTX2 potentially participates in immunoregulation in the tumor microenvironment. The knockdown of NPTX2 in HOS and SW1353 cells reduced cell proliferation, enhanced cell apoptosis, which demonstrates that NPTX2 may promote osteosarcoma progression. These findings suggest that NPTX2 can promote tumorigenesis and reduce the secretion of CCL4 in osteosarcoma tumor cells. Therefore, targeting NPTX2 may offer a novel approach for the immunotherapeutic management of osteosarcoma.

An accurate prognosis prediction model based on 11 *DEGs (MYCL*, *NDRG1*, *IFNLR1*, *C1GALT1C1L*, *ATP6V1G3*, *B3GALT4*, *MGAT1*, *PFN2*, *IL23A*, *ABCA7*, and *MAN2B1*) between hot and cold osteosarcomas was constructed and validated. MYCL is a promising treatment target for lung cancers.^43^ NDRG1, a downstream regulatory gene of N‐myc, inhibits metastasis, and recurrence of tumors such as gastric cancer.^44^ Over‐expression of B7‐H3 in colon cancer upregulates the B3GALT4 expression level. Therefore, B3GALT4 is a potential prognostic and treatment target for colon cancer.^45^ An in vitro study revealed that the overexpression of MGAT1 enhanced the proliferation and invasiveness of hepatocarcinoma cell lines.^46^ Profilin 2 (PFN2) binds and regulates the polymerization of actin.^47^ Immunotherapy has been shown to be an effective treatment for melanomas,^48^ non‐small cell lung cancer,^49^ bladder cancer,^50^ and several other cancers. PD‐L1 is an effective immunotherapy against numerous tumors that display immune infiltration.^51^ Using patients undergoing PD‐L1 therapy, we validated the accuracy of our model in predicting the prognosis of osteosarcoma patients. Despite these findings, this study is limited by the fact that the constructed model was only validated using one external dataset with limited data. Second, we did not assess the utility of the model in osteosarcoma patients receiving different therapies.

## 5 CONCLUSION

In summary, this study performed a comprehensive analysis of immune infiltrating cells in osteosarcoma. Upregulation of NPTX2 in cold tumor negatively correlated with the expression of CD8A, GZMB, and IFNG, and knockdown of NPTX2 increased the production of CCL4. In addition, in vitro experiments showed that NPTX2 expression influenced the biological behaviors of osteosarcoma cells. This suggests that NPTX2 disrupts immune response against osteosarcoma. Accordingly, NPTX2 is a potential immunotherapeutic target for osteosarcoma. Moreover, the 11 hub gene model can accurately predict the prognosis of osteosarcoma in patients undergoing immunotherapy.

## ETHICS STATEMENT

The protocol for this study was approved by the Ethics Committee of the First Affiliated Hospital of Zhengzhou University (2021‐KY‐0035). All participants consented to participate in this study. The research was conducted in line with the institutional guidelines.

## CONFLICT OF INTEREST

The authors declare no conflict of interest.

## Supporting information

Figure S1

Figure S2

Figure S3

## Data Availability

The data used in this study are available in the TCGA (http://tcga.cancer.gov/dataportal) and UCSCXena (https://xenabrowser.net/) portals.
