# Supplementary figures and images for: A comprehensive analysis of immune infiltration in the tumor microenvironment of osteosarcoma
Source: Cancer Med. 2021 Jul 13;10(16):5696–711. doi: 10.1002/cam4.4117 (PMC8366103; doi:10.1002/cam4.4117)

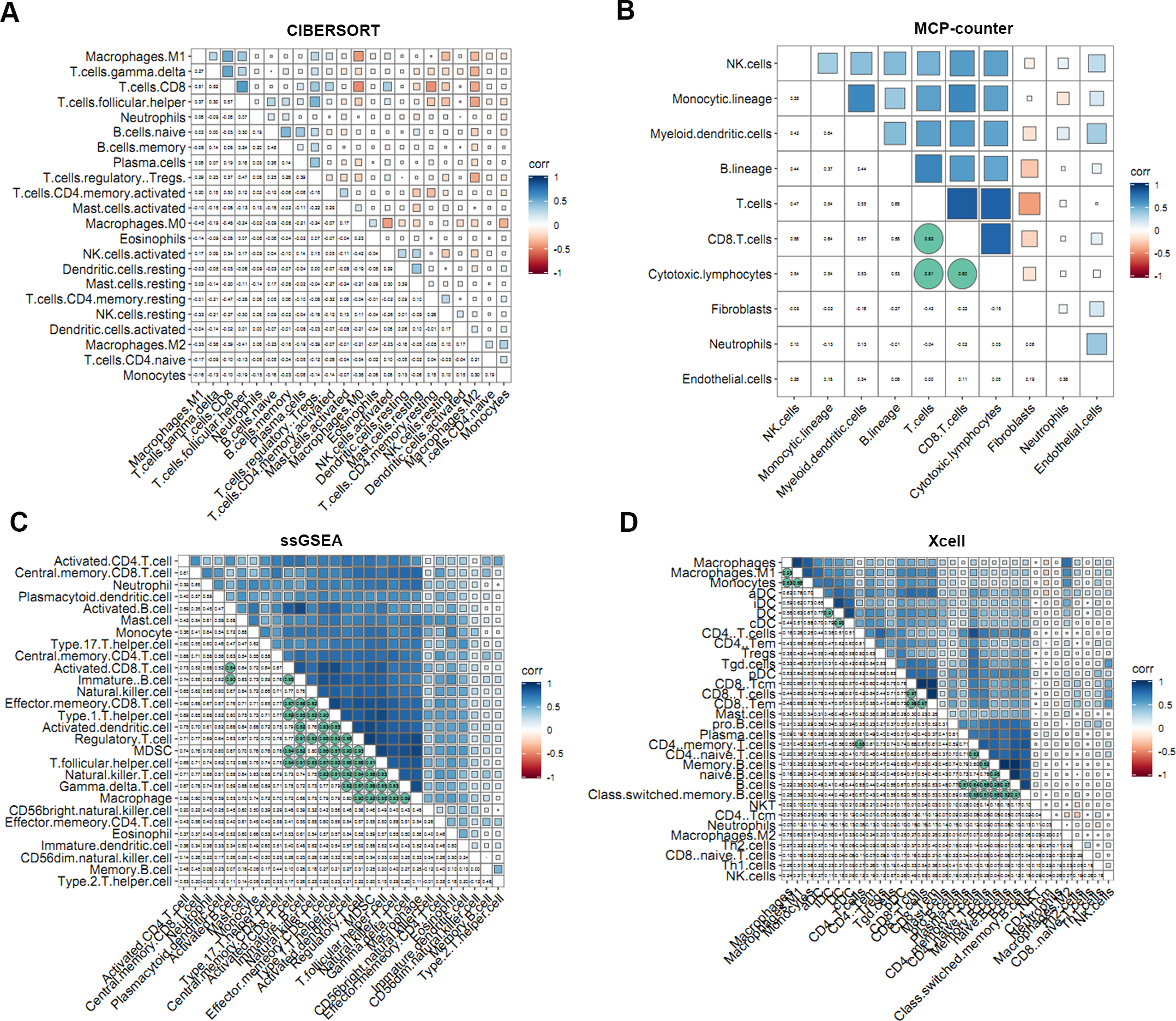

Supplement: Supplementary file 1 — Figure S1 [file CAM4-10-5696-s002.tif]

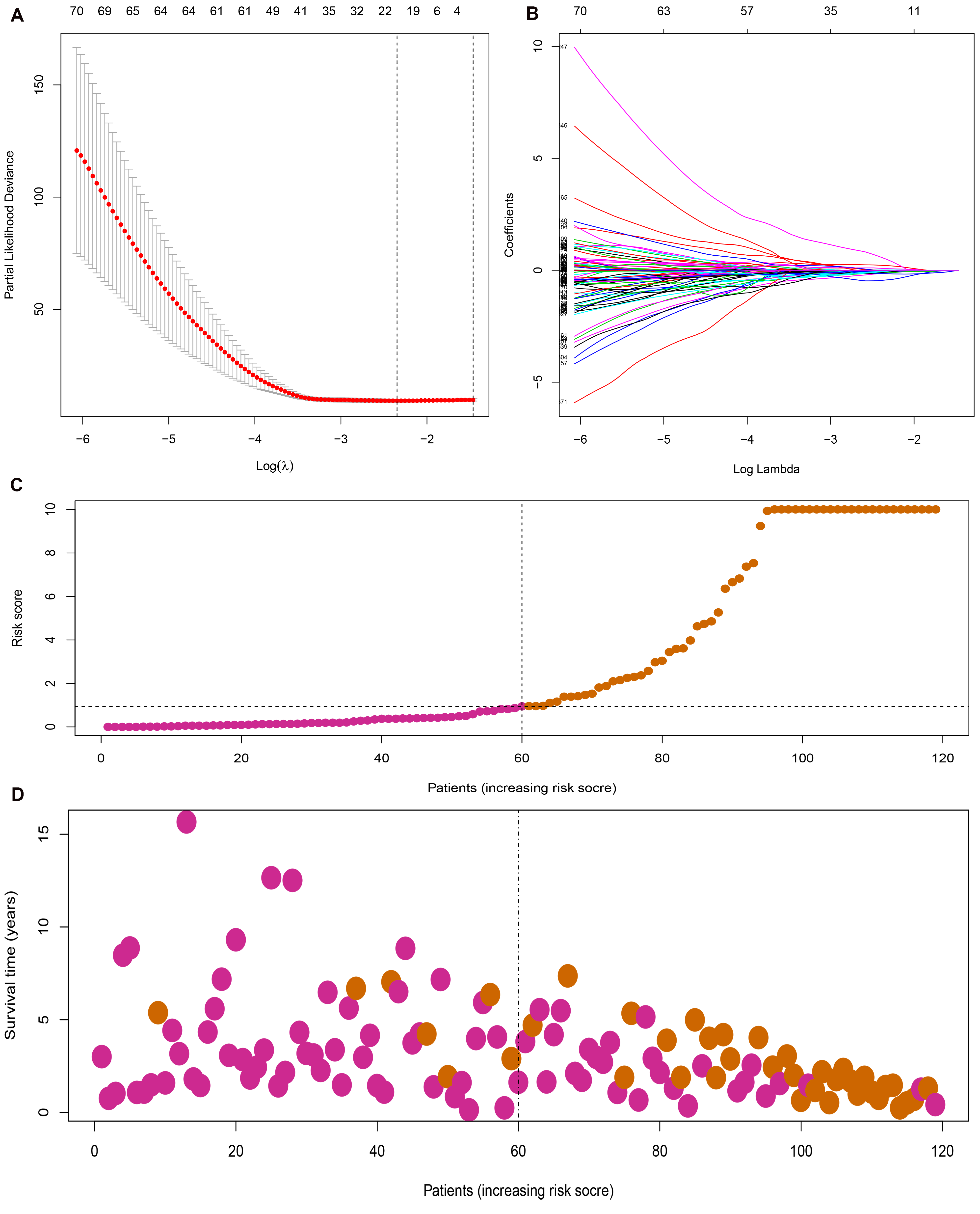

Supplement: Supplementary file 3 — Figure S3 [file CAM4-10-5696-s003.tif]
